# The Prognostic Value of Log Odds of Positive Lymph Nodes in Early-Stage Esophageal Cancer Patients: A Study Based on the SEER Database and a Chinese Cohort

**DOI:** 10.1155/2021/8834912

**Published:** 2021-03-05

**Authors:** Kexing Xi, Wenyou Chen, Hui Yu

**Affiliations:** ^1^Department of Colorectal Surgery and State Key Lab of Molecular Oncology, National Cancer Center/National Clinical Research Center for Cancer/Cancer Hospital, Chinese Academy of Medical Sciences and Peking Union Medical College, Beijing 100021, China; ^2^Department of Thoracic Surgery, The First Affiliated Hospital of Jinan University, Guangzhou 510630, China; ^3^Department of Thoracic Surgery, Sun Yat-sen University Cancer Center, Guangzhou 510060, China; ^4^State Key Laboratory of Oncology in South China, Collaborative Innovation Center for Cancer Medicine, Guangzhou 510060, China

## Abstract

**Objective:**

Early detection and timely treatment are important for improving the prognosis of esophageal cancer (EC). Identification of the prognostic risk factors could help us to discern the high-risk population. This study was aimed at exploring the prognostic significance of log odds of positive lymph nodes (LODDS) in early-stage EC patients.

**Methods:**

Patients who underwent esophagectomy and diagnosed as pathologic T1-2 N0 EC were reviewed between January 2005 and December 2015 from the Surveillance, Epidemiology, and End Results (SEER) database (the development cohort, *n* = 1004). The X-tile software was used to determine the optimal cutoff values of LODDS. A separate Chinese cohort including 245 patients (the validation cohort) was used to externally validate the results of the SEER database.

**Result:**

Patients were divided into two groups based on the cutoff points of LODDS: <−1.40 (LODDS1) and ≥−1.40 (LODDS2). In the development cohort, the 5-year overall survival (OS) rate was 75.3% for patients in the LODDS1 group, compared with 67.5% for those in the LODDS2 group (*P*=0.002). In multivariate Cox analysis, LODDS was associated with OS significantly (hazard ratio (HR), 1.48; 95% confidence intervals (CI), 1.19–1.85). In the validation cohort, the 5-year OS rate was 76.6% for patients in the LODDS1 group, compared with 64.4% for those in the LODDS2 group (*P*=0.006). The HR value in multivariate Cox analysis for OS was 2.00 (95% CI, 1.26–3.18).

**Conclusion:**

LODDS was an important independent factor for survival in early-stage EC patients.

## 1. Introduction

Esophageal cancer (EC) is one of the most common cancers and the fifth common cause of cancer-related death worldwide [1, 2]. The prognosis of EC is poor and the 5-year overall survival rate is approximately 17% for all patients [3]. Up to now, surgery is still the main treatment for early-stage and localized EC. However, surgical treatment remains unsatisfactory and the 5-year overall survival is less than 25% for patients who received surgery alone [4, 5]. The main cause of treatment failure for EC is local recurrence and distant metastasis.

Early detection and timely treatment are important for improving the prognosis of EC [6]. Identification of the prognostic risk factors is the first step which can help us to discern the high-risk population and take appropriate precautions. Moreover, for high-risk population, the postoperative follow-up should be more intensive so that there could be early detection of the recurrence and metastasis. TNM staging is significantly associated with the survival outcome for EC patients [6, 7]. Previous studies have reported that age, heavy drinking, smoking, etc. are also strong independent factors for EC [8–11]. Although many prognostic factors have been found, we still could not adequately stratify risk in EC patients. Therefore, we need to find additional new factors to provide more precise prognostic prediction for EC patients.

Recently, several studies have demonstrated that the logarithmic odds of positive lymph nodes (LODDS) plays an important role in some cancers for prognostic prediction, including colorectal cancer, gastric cancer, pancreatic cancer, and lung cancer [12–16]. LODDS is defined as the log of the ratio between the number of positive lymph nodes (PLNs) and the number of negative lymph nodes (NLNs). As a new prognostic factor, LODDS is considered better than the lymph node ratio (LNR, the number of PLNs divided by the number of examined LNs) and pN for predicting survival in some cancers [12]. LODDS takes into consideration the number of positive and negative lymph nodes (LNs), which may make it more precise than other factors for prognostic prediction for certain patients [15].

Therefore, in the present study, we specifically studied patients with T1-2 N0 M0 ESCC and determined the prognostic role of LODDS in patients with early-stage esophageal cancer.

## 2. Materials and Methods

### 2.1. Patient Selection and Data Collection

**Figure 1:**
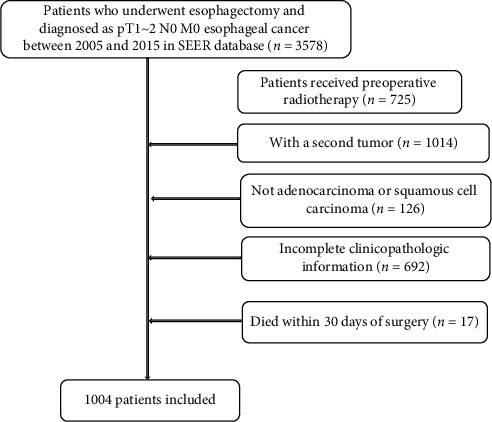
The selection flow diagram of patients in the SEER database.

We reviewed consecutive esophageal cancer patients who underwent esophagectomy between January 2005 and December 2015 from the Surveillance, Epidemiology, and End Results (SEER) database. Patients were included based on the following criteria: (1) patients who underwent esophagectomy, confirmed R0 resection; (2) diagnosed as pT1-2 N0 M0 esophageal cancer. The excluding criteria were as follows: (1) patients who received preoperative radiotherapy; (2) with a second cancer; (3) not adenocarcinoma or squamous cell carcinoma; (4) incomplete clinicopathologic information; (5) patients who were less than 18 years of age; and (6) patients who died within 30 days of surgery. Finally, 1004 patients in SEER database were included in this retrospective study (Figure 1).

To validate the results of the SEER databases, EC patients who underwent esophagectomy at Sun Yat-sen University Cancer Center in Guangzhou (Guangdong, China) between January 2005 and June 2010 were selected under the same criteria of exclusions and inclusions which were used in the SEER database. Moreover, patients who had received preoperative chemotherapy or chemoradiotherapy were also excluded. Finally, 245 patients were included for further analysis. This study was approved by the Institute Research Medical Ethics Committee of Sun Yat-sen University Cancer Center. Informed consent was obtained from all patients before the study. Patients of the Chinese cohort had follow-up after surgery every 3 months for the first year, every 6 months for the second to third year, and once a year thereafter. Evaluations included tumor markers, endoscopy with or without biopsy, thoracoabdominal CT, and esophageal barium swallow generally. The follow-up data was reviewed by July 1, 2015.

### 2.2. Statistical Analysis

The optimal cutoff values of LODDS were identified using the X-tile software (Version 3.6.1, Copyright Yale University 2003). We used *χ*^2^ or Fisher's exact tests to compare the baseline characteristics between different LODDS groups. We compared the differences of survival using Kaplan–Meier method and log-rank test. Univariate Cox regression was applied to calculate hazard ratio (HR) for continuous variables. The variables which were significant in the univariate analysis were tested by using multivariate analysis. In the development cohort, age, tumor size, tumor location, pathologic *T* (pT) status, histology, adjuvant radiotherapy, number of resected LNs, and LODDS were included for multivariate analysis. And Cox regression multivariate analysis was used to test the variables: pT status, number of resected LNs, and LODDS in the validation cohort. Statistical analyses were completed using SPSS 25.0 software (SPSS Inc., Chicago, IL, USA). *P* value less than 0.05 was considered statistically significant. LODDS was calculated as log ((the number of PLNs + 0.5)/(the number of NLNs + 0.5)); 0.5 is added to both the numerator and the denominator to avoid an infinite number [14, 17, 18].

## 3. Results

### 3.1. Patient Characteristics

**Figure 2:**
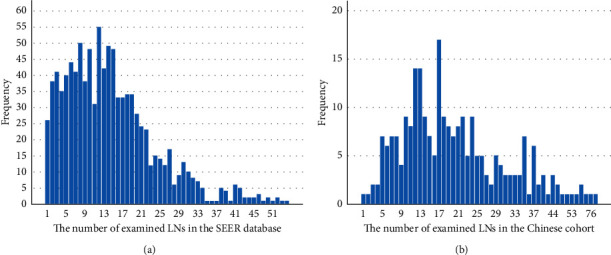
Distribution of the number of examined LNs in the development cohort and validation cohort.

Patients with T1-2 N0 M0 esophageal cancer (EC) were enrolled in the present study, including 1004 patients from the SEER database (development cohort) and 245 patients from a Chinese cohort (validation cohort). The demographics and tumor characteristics of all patients are summarized in Table 1. The distribution of the number of resected LNs in patients is shown in Figure 2. The mean and median numbers of resected LNs in the SEER database were 14.5 and 13.0, respectively (range, 1–71). In the Chinese cohort, the mean and median numbers of resected LNs were 20.6 and 18.0, respectively (range, 1–79).

### 3.2. The Optimal Cutoff Points of LODDS

**Figure 3:**
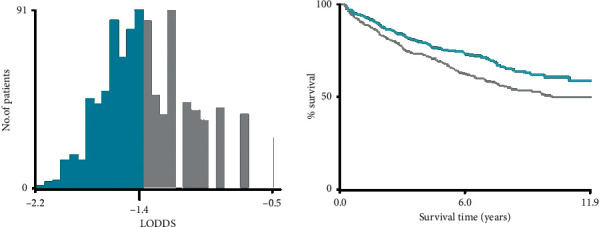
X-tile analysis for the optimal cutoff values of LODDS.

According to the results of the X-tile, the optimal cutoff points of LODDS were identified as < −1.40 (LODDS1) and ≥−1.40 (LODDS2) based on the patients of the SEER database (development cohort) (Figure 3). Then, patients in the Chinese cohort (validation cohort) were also divided into two groups according to the above cutoff points of LODDS. The correlations between baseline characteristics and LODDS groups are shown in Table 1. Only surgical approach was significantly associated with LODDS in the validation cohort (*P*=0.002).

### 3.3. Prognostic Significance of LODDS on Survival

**Figure 4:**
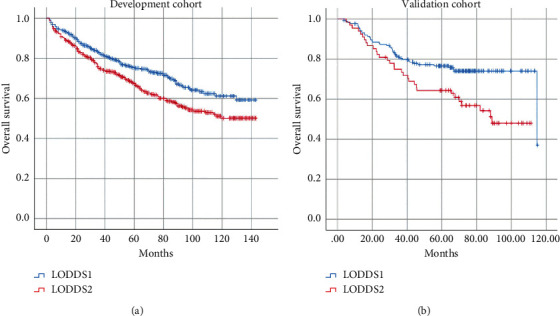
(a) Kaplan–Meier curves for overall survival of 1004 EC patients in the development cohort. (b) Kaplan–Meier curves for overall survival of 245 EC patients in the validation cohort.

First, we used Kaplan–Meier curves to evaluate the association between LODDS and overall survival (OS) in the development cohort. The 5-year OS rate was 75.3% for patients in LODDS1 group, compared with 67.5% for those in LODDS2 group (*P*=0.002) (Figure 4(a)). In the validation cohort, the 5-year OS rate was 76.6% for patients in LODDS1 group, compared with 64.4% for those in LODDS2 group (*P*=0.006) (Figure 4(b)).

Cox univariate and multivariate analyses were performed to find the most significant prognostic factors of OS. In the development cohort, age, tumor size, tumor location, pT status, histology, adjuvant radiotherapy, number of resected LNs, and LODDS were significantly associated with OS in univariate analysis. Moreover, age, pT status, histology, and LODDS were found to be independent prognostic factors for OS in multivariate analysis (all *P* values <0.001). Both in univariate and multivariate Cox analyses, LODDS was associated with OS significantly (hazard ratio (HR), 1.42; 95% confidence intervals (CI), 1.14–1.77 and HR, 1.48; 95% CI, 1.19–1.85, respectively) (Table 2).

In the validation cohort, the univariate analysis revealed that pT status, number of resected LNs, and LODDS were significantly related to OS (*P*=0.023, 0.009, and 0.007, respectively). Then, Cox multivariate analysis demonstrated that pT status and LODDS were significant prognostic factors of OS (*P*=0.012 and 0.003, respectively). The HR values in univariate and multivariate Cox analyses for OS were 1.89 (95% CI, 1.19–3.00) and 2.00 (95% CI, 1.26–3.18), respectively (Table 3). Therefore, LODDS was an independent prognostic factor of OS in the development cohort and validation cohort.

## 4. Discussion

In this present study, we used a relatively new statistical marker, LODDS, to evaluate the prognostic value for early EC patients and we found that LODDS was an important factor of survival. Several lines of evidence have demonstrated that LNs status is one of the most important risk factors of survival for esophageal cancer (EC) [19–21]. The current pN staging of the International Union Against Cancer (UICC) is based mainly on the number of metastatic LNs. Although the current guidelines play an important role in establishing the pN categories, however, there exist some problems; for example, the TNM staging system could not provide more meaningful information for node-negative patients. It also can lead to the phenomenon of stage migration [22].

Recently, researchers have tried to find other factors to predict the prognosis of EC and improve the efficacy of pN categories, such as the LNR (positive lymph node ratio). However, it could not provide more information for pN0 patients compared with TNM. For example, the LNRs of patients with T1-2 N0 M0 are all zero. LODDS, as another new prognostic factor, takes into consideration the number of PLNs and NLNs, which may become a better factor for predicting prognosis.

In the present study, we retrospectively evaluated the prognostic role of LOODS in T1-2 N0 M0 EC. We found that LODDS was significantly associated with OS in both the development and validation cohort. The survival was significantly better in the LODDS1 group than that in the LODDS2 group; the prognosis significantly decreased with the increased LODDS value. The above results were consistent with previous studies [18]. In this study, the enrolled EC patients were all node-negative, and the results showed that LODDS played an important role in predicting survival outcome for EC patients. Compared with the pN staging, it could provide additional meaningful information for node-negative patients.

Several studies had demonstrated that LODDS was more accurate to predict survival in some solid tumors, such as colon cancer and gastric cancer [23, 24]. LODDS contained information, both PLN and NLN. And previous studies had shown that the number of NLNs was related to the survival in EC patients [25]. However, few studies explored the role of LODDS in survival of early-stage EC patients. In this research, we specially studied T1-2 N0 M0 ESCC patients and the results confirmed that LODDS was a strong independent factor for early-stage EC patients. Although previous studies had shown the prognostic superiority of LODDS compared with LNR, pN staging in several solid carcinomas including EC [16, 18], it still needed further validation.

In this study, we firstly determined the cutoff values of LODDS based on the SEER database. Then, a separate Chinese cohort was used to externally validate the results of the SEER database. Few studies had done it like this. Moreover, the SEER database is based on the US population. All of this makes our results more reliable and representative.

There were some limitations in the present study. Firstly, this was a retrospective study, which could bring the bias. Secondly, As the cutoff values varied among different researches, whether our results and the cutoff values of LODDS could be applied to other institutions remain to be further proved. Thirdly, the missing data on clinicopathologic factors, limited treatment data in the SEER database which could result in bias, such as the radiotherapy field design and dose, and chemotherapy regimen and dose could influence survival outcome.

In conclusion, our study demonstrated that LODDS was an important independent factor for survival in early-stage EC patients. The results of this study might provide additional information for survival outcome of early-stage EC patients.

## Figures and Tables

**Table 1:** The correlation between LODDS and patient characteristics in the SEER database (development cohort) and the Chinese cohort (validation cohort).

Characteristics	Development cohort *n* (%)				Validation cohort *n* (%)			
	Total	LODDS1	LODDS2	*P* value	Total	LODDS1	LODDS2	*P* value
Gender				0.115				0.621
Male	845 (84.2)	426 (82.4)	419 (86.0)		182 (74.3)	133 (75.1)	49 (72.1)	
Female	159 (15.8)	91 (17.6)	68 (14.0)		63 (25.7)	44 (24.9)	19 (27.9)	

Age				0.447				0.428
≦65 years	571 (56.9)	300 (58.0)	271 (55.6)		192 (78.4)	141 (79.7)	51 (75.0)	
>65 years	433 (43.1)	217 (42.0)	216 (44.4)		53 (21.6)	36 (20.3)	17 (25.0)	

Smoking status								0.850
Never					96 (39.2)	70 (39.5)	26 (38.2)	
Former					149 (60.8)	107 (60.5)	42 (61.8)	

Alcohol consumption								0.506
No					201 (82.0)	147 (83.1)	54 (79.4)	
Yes					44 (18.0)	30 (16.9)	14 (20.6)	

Tumor size (cm)				0.102				0.922
≦3	640 (63.7)	320 (61.9)	320 (65.7)		144 (58.8)	104 (58.8)	40 (58.8)	
≦5	109 (10.9)	64 (12.4)	45 (9.2)		68 (27.8)	50 (28.2)	18 (26.5)	
>5	45 (4.5)	29 (5.6)	16 (3.3)		33 (13.5)	23 (13.0)	10 (14.7)	
Unknown	210 (20.9)	104 (20.1)	106 (21.8)		0 (0)	0 (0)	0 (0)	

Tumor location				0.244				0.333
Upper	22 (2.2)	9 (1.7)	13 (2.7)		34 (13.9)	28 (15.8)	6 (8.8)	
Middle	119 (11.9)	69 (13.3)	50 (10.3)		154 (62.9)	110 (62.1)	44 (64.7)	
Lower	757 (75.4)	390 (75.4)	367 (75.4)		57 (23.3)	39 (22.0)	18 (26.5)	
Unknown	106 (10.6)	49 (9.5)	57 (11.7)		0 (0)	0 (0)	0 (0)	

pT status				0.571				0.250
T1	830 (82.7)	424 (82.0)	406 (83.4)		101 (41.2)	69 (39.0)	32 (47.1)	
T2	174 (17.3)	93 (18.0)	81 (16.6)		144 (58.8)	108 (61.0)	36 (52.9)	

Differentiation				0.137				0.162
Well	182 (18.1)	93 (18.0)	89 (18.3)		62 (25.3)	39 (22.0)	23 (33.8)	
Moderate	446 (44.4)	236 (45.6)	210 (43.1)		112 (45.7)	84 (47.5)	28 (41.2)	
Poor/undifferentiated	252 (25.1)	136 (26.3)	116 (23.8)		71 (29.0)	54 (30.5)	17 (25.0)	
Unknown	124 (12.4)	52 (10.1)	72 (14.8)		0 (0)	0 (0)	0 (0)	

Histology				0.343				0.066
Adenocarcinoma	857 (85.4)	436 (84.3)	421 (86.4)		4 (1.6)	176 (99.4)	65 (95.6)	
Squamous cell carcinoma	147 (14.6)	81 (15.7)	66 (13.6)		241 (98.4)	1 (0.6)	3 (4.4)	

Surgical approach								0.002
Sweet					165 (67.3)	109 (61.6)	56 (82.4)	
Ivor-lewis/Mckeown					80 (32.7)	68 (38.4)	12 (17.6)	

Anastomosis								0.228
Hand-sewn					36 (14.7)	29 (16.4)	7 (10.3)	
Stapled					209 (85.3)	148 (83.6)	61 (89.7)	

LODDS: log odds of positive lymph nodes.

**Table 2:** Cox univariate and multivariate analyses of prognostic factors for overall survival in the development cohort.

Variable	Univariate analysis		Multivariate analysis	
	HR (95% CI)	*P*	HR (95% CI)	*P*
Gender		0.071		
Male	Reference			
Female	1.30 (0.98–1.73)			

Age (years)		<0.001		<0.001
≤65	Reference		Reference	
＞65	1.72 (1.38–2.13)		1.66 (1.34–2.06)	

Tumor size (cm)		0.009		
≦3	Reference			
≦5	1.41 (1.02–1.96)	0.040		
>5	1.58 (1.00–2.50)	0.051		
Unknown	0.81 (0.61–1.08)	0.143		

Tumor location		<0.001		
Upper	Reference			
Middle	1.47 (0.73–2.97)	0.278		
Lower	0.77 (0.39–1.49)	0.435		
Unknown	0.82 (0.39–1.71)	0.593		

pT status		<0.001		<0.001
T1	Reference		Reference	
T2	1.97 (1.54–2.52)		1.80 (1.40–2.31)	

Differentiation		0.059		
Well	Reference			
Moderate	1.23 (0.89–1.71)	0.203		
Poor/undifferentiated	1.48 (1.05–2.09)	0.025		
Unknown	0.98 (0.64–1.50)	0.915		

Histology		<0.001		<0.001
Adenocarcinoma	Reference		Reference	
Squamous cell carcinoma	2.20 (1.70–2.86)		2.01 (1.54–2.63)	

Adjuvant radiotherapy		0.002		
No	Reference			
Yes	2.07 (1.30–3.29)			

Number of resected LNs		0.003		
<15	Reference			
≧15	0.71 (0.56–0.89)			

LODDS		0.002		<0.001
LODDS1	Reference		Reference	
LODDS2	1.42 (1.14–1.77)		1.48 (1.19–1.85)	

Age, tumor size, tumor location, pT status, histology, adjuvant radiotherapy, number of resected LNs, and LODDS were included for multivariate analysis. HR: hazard ratio; CI: confidence interval; LODDS: log odds of positive lymph nodes; LN: lymph node.

**Table 3:** Cox univariate and multivariate analyses of prognostic factors for overall survival in the validation cohort.

Variable	Univariate analysis		Multivariate analysis	
	HR (95% CI)	*P*	HR (95% CI)	*P*
Gender		0.250		
Male	Reference			
Female	1.34 (0.82–2.19)			

Age (years)		0.505		
≤65	Reference			
>65	1.19 (0.71–2.01)			

Smoking status		0.909		
Never	Reference			
Former	1.03 (0.64–1.64)			

Alcohol consumption		0.086		
No	Reference			
Yes	1.60 (0.94–2.72)			

Tumor size (cm)		0.517		
≦3	Reference			
≦5	1.32 (0.79–2.20)	0.290		
>5	1.28 (0.66–2.50)	0.469		

Tumor location		0.519		
Upper	Reference			
Middle	1.59 (0.72–3.50)	0.254		
Lower	1.55 (0.65–3.70)	0.329		

pT status		0.023		0.012
T1	Reference		Reference	
T2	1.77 (1.08–2.89)		1.88 (1.15–3.07)	

Differentiation		0.134		
Well	Reference			
Moderate	1.20 (0.66–2.20)	0.552		
Poor/undifferentiated	1.80 (0.97–3.34)	0.064		

Histology		0.215		
Adenocarcinoma	Reference			
Squamous cell carcinoma	0.41 (0.10–1.68)			

Adjuvant therapy		0.304		
No	Reference			
Yes	0.36 (0.05–2.56)			

Surgical approach		0.291		
Sweet	Reference			
Ivor-lewis/Mckeown	0.76 (0.46–1.26)			

Anastomosis		0.917		
Hand-sewn	Reference			
Stapled	1.04 (0.55–1.96)			

Number of resected LNs		0.009		
<15	Reference			
≥15	0.55 (0.35–0.86)			

LODDS		0.007		0.003
LODDS1	Reference		Reference	
LODDS2	1.89 (1.19–3.00)		2.00 (1.26–3.18)	

HR: hazard ratio, CI: confidence interval, LODDS: log odds of positive lymph nodes, LN: lymph node. pT status, number of resected LNs, and LODDS were included for multivariate analysis.

## Data Availability

The authors may balance the potential benefits and risks for each request and then provide the data that could be shared.
